# Prophylactic, preemptive, and curative treatment for sinusoidal obstruction syndrome/veno-occlusive disease in adult patients: a position statement from an international expert group

**DOI:** 10.1038/s41409-019-0705-z

**Published:** 2019-10-01

**Authors:** Mohamad Mohty, Florent Malard, Manuel Abecasis, Erik Aerts, Ahmed S. Alaskar, Mahmoud Aljurf, Mutlu Arat, Peter Bader, Frederic Baron, Grzegorz Basak, Ali Bazarbachi, Didier Blaise, Fabio Ciceri, Selim Corbacioglu, Jean-Hugues Dalle, Fiona Dignan, Takahiro Fukuda, Anne Huynh, Jurgen Kuball, Silvy Lachance, Hillard Lazarus, Tamas Masszi, Mauricette Michallet, Arnon Nagler, Mairead NiChonghaile, Shinichiro Okamoto, Antonio Pagliuca, Christina Peters, Finn B. Petersen, Paul G. Richardson, Tapani Ruutu, Wael Saber, Bipin N. Savani, Robert Soiffer, Jan Styczynski, Elisabeth Wallhult, Ibrahim Yakoub-Agha, Rafael F. Duarte, Enric Carreras

**Affiliations:** 1 Hematology Department, AP-HP, Hôpital Saint-Antoine, Sorbonne Université, Paris, France; 2 Instituto Portugues de Oncologia, Lisbon, Portugal; 3 Department of Internal Medicine-Oncology, University Hospital Zurich, Zurich, Switzerland; 4 Department of Oncology, King Abdulaziz Medical City, King Abdullah International Medical Research Center, King Saud Bin Abdulaziz University for Health Sciences, Ministry of National Guard Health Affairs, Riyadh, Saudi Arabia; 5 King Faisal Specialist Hospital and Research Centre, Riyadh, Saudi Arabia; 6 HSCT Unit, Sisli Florence Nightingale Hospital, Istanbul, Turkey; 7 Division for Stem Cell Transplantation and Immunology, Department for Children and Adolescents, University Hospital, Goethe University, Frankfurt/Main, Germany; 8 Department of Hematology, University of Liege, Liege, Belgium; 9 Medical University of Warsaw, Warsaw, Poland; 10 Department of Internal Medicine, American University of Beirut, Beirut, Lebanon; 11 Transplant and Cellular Immunotherapy Program, Department of Hematology, Aix-Marseille Univ, Inserm, CNRS, Institut Paoli-Calmettes, CRCM, Marseille, France; 12 Hematology and Bone Marrow Transplantation Unit, IRCCS Ospedale San Raffaele, University Vita-Salute, Milano, Italy; 13 Department of Pediatric Hematology, Oncology and Stem Cell Transplantation, University of Regensburg, Regensburg, Germany; 14 Department of Hematology and Immunology, Hospital Robert Debre, Paris 7-Paris Diderot University, Paris, France; 15 Department of Clinical Haematology, Central Manchester Foundation Trust, Manchester, UK; 16 HSCT Division, National Cancer Center Hospital, Tokyo, Japan; 17 Hematology Department, Institut Universitaire du Cancer Toulouse–Oncopole, Toulouse, France; 18 Department of Haematology, University Medical Centre, Utrecht, The Netherlands; 19 Department of Hematology and Stem Cell Transplant Program, Hôpital Maisonneuve-Rosemont, University of Montreal, Montreal, QC Canada; 20 Case Western Reserve University, Cleveland, OH USA; 21 Department of Hematology and Stem Cell Transplantation, St. Istvan and St. Laszlo Hospital, Budapest 3 Department of Internal Medicine, Semmelweis University, Budapest, Hungary; 22 Hematology Department, Groupement Hospitalier Sud, Hospices Civils de Lyon, Université Claude Bernard Lyon EST, Pierre Bénite, France; 23 Hematology and Bone Marrow Transplantation, Chaim Sheba Medical Center, Tel-Hashomer, Israel; 24 National Stem Cell Transplant Unit (Adults), Department of Haematology, St James’s Hospital and Academic Department of Haematology, Trinity College Dublin, Dublin, Ireland; 25 Division of Hematology, Department of Medicine, Keio University School of Medicine, Tokyo, Japan; 26 Department of Haematological Medicine, King’s College Hospital, London, UK; 27 Department of Pediatrics, St. Anna Kinderspital, 1090 Vienna, Austria; 28 LDS Hospital, Salt Lake City, UT USA; 29 Division of Hematology, Mayo Clinic, Rochester, MN USA; 30 Clinical Research Institute, Helsinki University Hospital, Helsinki, Finland; 31 Center for International Blood and Marrow Transplant Research, Medical College of Wisconsin, Milwaukee, WI USA; 32 Hematology and Stem Cell Transplantation Section, Division of Hematology/Oncology, Department of Medicine, Vanderbilt University Medical Center and Veterans Affairs Medical Center, Nashville, TN USA; 33 Division of Hematologic Malignancies, Dana-Farber Cancer Institute, Boston, MA USA; 34 Pediatric Hematology and Oncology, University Hospital, Collegium Medicum UMK, Bydgoszcz, Poland; 35 Section of Haematology and Coagulation, Department of Internal Medicine, Sahlgrenska University Hospital, Göteborg, Sweden; 36 CHU de Lille, LIRIC INSERM U995, Université de Lille2, Lille, France; 37 Department of Hematology, Hospital Universitario Puerta de Hierro Majadahonda, Madrid, Spain; 38 Hematology Department, Josep Carreras Foundation and Leukemia Research Institute, Hospital Clínic, Barcelona, Spain

**Keywords:** Haematological diseases, Therapeutics

## Abstract

Sinusoidal obstruction syndrome, also known as veno-occlusive disease (SOS/VOD), is a potentially life-threatening complication that can develop after hematopoietic cell transplantation (HCT). While SOS/VOD may resolve within a few weeks in the majority of patients with mild-to-moderate disease, the most severe forms result in multiorgan dysfunction and are associated with a high mortality rate (>80%). Therefore, careful surveillance may allow early detection of SOS/VOD, particularly as the licensed available drug is proven to be effective and reduce mortality. The aim of this work is to propose an international consensus guideline for the treatment and prevention of SOS/VOD in adult patients, on behalf of an international expert group.

## Introduction

Sinusoidal obstruction syndrome (SOS), formerly called veno-occlusive disease (VOD; referred to as SOS/VOD hereafter), is a life-threatening complication that can occur after hematopoietic stem cell transplantation (HCT) [1]. The conditioning regimen and immune-mediated injury following allogeneic HCT (allo-HCT) generate toxic metabolites that damage sinusoidal endothelial cells. The expression of tissue and von Willebrand factors contribute to the clothing cascade activation, perpetuate the endothelial cell injury leading to the formation of gaps in the hepatic sinusoidal endothelium [2]. Red blood cells penetrate through those gaps in the perisinusoidal space, beneath the endothelial cells, and subsequently dissect off the endothelial lining, all of which embolize as part of the sinusoid flow and in turn obstruct the sinusoid [1]. This process reduce hepatic outflow, produces postsinusoidal hypertension with tissue ischemia in zone 3 of the acinus, and concomitant hepatocellular damage; all of which results in the clinical symptoms of SOS/VOD and an associated hepato-renal syndrome, namely fluid retention, ascites, weight gain, painful hepatomegaly, and jaundice [3–5]. In the most severe cases, patients may develop multiorgan dysfunction (MOD) with pulmonary and renal involvement, encephalopathy and, ultimately, death. Despite the incidence of SOS/VOD being limited, around 10–15% after myeloablative allo-HCT and up to 5% after reduced-intensity conditioning (RIC) allo-HCT, particular attention must be paid to permit its early detection and treatment and to prevent the development of the most severe forms, which are in turn associated with a very high mortality rate (>80%) [1, 5]. In an effort to improve early diagnosis, the European Group for Blood and Marrow Transplantation (EBMT) revise the modified Seattle [6] and Baltimore [4] criteria, and recently published revised diagnosis and severity criteria for adults [7].

The next step was to provide treatment recommendation for SOS/VOD in adult patients. Commissioned by the EBMT, a global SOS/VOD Task Force was developed to help identify and address the key challenges in the prophylactic, preemptive, and curative treatment for SOS/VOD in adult patients. The Task Force committee met in 2018 and identified five key clinical practice questions (risk factors, how to treat, when to treat, supportive care, and preventive therapy) relevant to clinical hematologists and allied health practitioners. Practice guidelines from this initiative for each particular question forms the basis of this article and lays out a roadmap of common issues encountered with prophylactic, preemptive, and curative treatment for SOS/VOD in adult patients with reference to the commercially available products and on clinical trials.

## EBMT diagnosis and severity criteria for SOS/VOD

In adult patients, Baltimore criteria have been reported to be more specific than the Seattle one for SOS/VOD diagnosis: specifically, while hemodynamic studies could not confirm the diagnosis in 42% of patients assessed by the Seattle criteria, such lack of confirmation was seen in only 9% of patients using the Baltimore criteria, a finding further validated by corroboration with histopathology [8, 9]. Coppell et al.’s meta-analysis yielded an almost 100% discrepancy in the incidence of SOS/VOD (Baltimore 9.6% vs. Seattle 17.3%) [5] and Yakushijin et al. report an even higher difference of 2.5% with the Baltimore criteria vs. 10.8% with the modified Seattle criteria [10]. This discrepancy between both classifications was related to hyperbilirubinemia, mandatory in the Baltimore, but not in the Seattle criteria. However, hyperbilirubinemia and jaundice are almost invariably present in classic SOS/VOD in adult patients [8]. Therefore, it was decided to keep the Baltimore criteria for diagnosis of classical SOS/VOD (within 21 days after HCT) in the revised EBMT criteria (Table 1) [7]. In contrast, beyond day 21, hyperbilirubinemia is less consistent [8, 11, 12]. Therefore, hyperbilirubinemia is not mandatory for the diagnosis of late SOS/VOD, provided patients present with at least two other clinical manifestations (painful hepatomegaly, weight gain >5%, and/or ascites) as well as hemodynamic and/or ultrasound evidence of SOS/VOD. In adult patients, thrombocytopenia with platelet transfusion refractoriness was not retained as a criterion, given the frequency and the lack of specificity of this symptom in the pancytopenic phase after allo-HCT [7].

**Table 1:** EBMT criteria for SOS/VOD diagnosis in adults

Classical SOS/VOD	Late-onset SOS/VOD
In the first 21 days after HSCT	>21 days after HSCT
Bilirubin ≥ 2 mg/dL and two of the following criteria must be present: - Painful hepatomegaly - Weight gain > 5% - Ascites	Classical VOD/SOS beyond day 21 OR Histologically proven SOS/VOD OR Two or more of the following criteria must be present: - Bilirubin ≥ 2 mg/dL (or 34 μmol/L) - Painful hepatomegaly - Weight gain > 5% - Ascites AND hemodynamical or/and ultrasound evidence of SOS/VOD

These symptoms/signs should not be attributable to others causes

Simultaneously, the EBMT proposed criteria for severity grading of SOS/VOD once the diagnosis is made [7]. SOS/VOD is graded in four stages of severity (mild, moderate, severe, and very severe), based on five parameters: time since first clinical manifestation of SOS/VOD, bilirubin level and kinetics, transaminase level, weight gain, and renal function (Table 2). Importantly, in the presence of two or more risk factors, patients are classified in the upper grade. Yoon et al. have validated these criteria in 203 patients with SOS/VOD [13]: 5.9% were in the mild, 12.8% moderate, 18.2% severe, and the majority (63.1%) was in the very severe grade. The day 100 overall survival (OS) of mild, moderate, severe, and very severe groups was 83.3, 84.3, 94.6, and 58.6%, respectively, and very severe SOS/VOD showed a significantly lower OS than the others (58.6 vs. 89.3%, *p* < 0.0001). Similarly, the day 100 transplant-related mortality was significantly higher in very severe SOS/VOD, being 36.7, vs. 8.3% in mild, 8.0% in moderate, and 2.7% in severe (*p* < 0.0001). This study confirms the worse outcome of very severe SOS/VOD, while severe SOS/VOD seems to have similar outcome to mild and moderate SOS/VOD. Overall further validation might be required with careful evaluation of therapeutic intervention to evaluate the prognosis of severe SOS/VOD in the defibrotide (DF) era.

**Table 2:** EBMT criteria for severity grading of a suspected SOS/VOD in adults

	Mild^a^	Moderate^a^	Severe	Very severe MOD/MOF^b^
Time since first clinical symptoms of SOS/VOD^c^	>7 days	5–7 days	≤4 days	Any time
Bilirubin (mg/dL)	≥2 and <3	≥3 and <5	≥5 and <8	≥8
Bilirubin (μmol/L)	≥34 and <51	≥51 and <85	≥85 and <136	≥136
Bilirubin kinetics			Doubling within 48 h	
Transaminases	≤2 × normal	>2 and ≤5 × normal	>5 and ≤8 × normal	>8 × normal
Weight increase	<5%	≥5% and <10%	≥5 % and <10%	≥10%
Renal function	<1.2 × baseline at transplant	≥1.2 and <1.5 × baseline at transplant	≥1.5 and <2 × baseline at transplant	≥2 × baseline at transplant or others signs of MOD/MOF

Patients belong to the category that fulfills two or more criteria. If patients fulfill two or more criteria in two different categories, they must be classified in the most severe category. Patient’s weight increase ≥5% and <10% is considered by default as a criterion for severe SOS/VOD, however if patients do not fulfill other criteria for severe SOS/VOD, weight increase ≥5 % and <10% is therefore considered as a criterion for moderate SOS/VOD

^a^In the case of the presence of two or more risk factors for SOS/VOD, patients should be in the upper grade

^b^Patients with multiorgan dysfunction must be classified as very severe

^c^Time from the date when the first signs/symptoms of SOS/VOD began to appear (retrospectively determined) and the date when the symptoms fulfilled SOS/VOD diagnostic criteria

## Risk factors

Given the role of risk factors in the severity grading of SOS/VOD (patients with two or more risk factors are classified in the upper grade), an accurate identification of these high-risk factors must be performed, as previously discussed (Table 3) [7]. Here, we highlight several important issues regarding risk factor evaluation. For haploidentical donors, studies using the Baltimore RIC regimen and posttransplant cyclophosphamide protocol showed no increased incidence of SOS/VOD [14, 15]. However, this may not be the case in the context of haploidentical allo-HCT with other conditioning regimens incorporating one or more alkylating agents and posttransplant cyclophosphamide. We therefore maintain the previous recommendation to consider haploidentical donor as a risk factor as any HLA-mismatched donor. The increased risk of SOS/VOD associated with inotuzumab ozogamicin has been recently confirmed, with an incidence of 11 vs. 1% with standard chemotherapy, in a prospective randomized phase-3 trial in relapse/refractory acute lymphoblastic leukemia [16]. In particular, specific warning is recommended when allo-HCT is planned shortly after inotuzumab ozogamicin salvage treatment for relapsed ALL. Similar risks are reported in clinical practice after pegylated asparaginase recent treatment with documented severe liver toxicity [17]. We also raise the question of the impact of immune checkpoint inhibitors (anti-CTLA4, anti-PD1, and anti-PDL1 monoclonal antibodies) on the risk of SOS/VOD. Autoimmune complications, including hepatitis, are frequent and well reported [18], translating in an increased risk of graft-vs.-host disease (GVHD) when they are used in the setting of allo-HCT [19–22]. In contrast, no formal study has so far reported an increased incidence of SOS/VOD in this setting [19–22]. Therefore, immune checkpoint inhibitors should not be considered for the moment as a confirmed risk factor of SOS/VOD.

**Table 3:** Risk factors for SOS/VOD

Transplant-related factors
Unrelated donor
HLA-mismatched donor
Non-T-cell depleted transplant
Myeloablative conditioning regimen
Oral or high-dose busulfan-based regimen
High-dose TBI-based regimen
Second HSCT
Patient and disease related factors
Older age
Karnofsky score below 90%
Metabolic syndrome
Female receiving norethisterone
Advanced disease (beyond second CR or relapse/refractory)
Thalassemia
Genetic factors (GSTM1 polymorphism, C282Y allele, *MTHFR* 677CC/1298CC haplotype)
Hepatic related
Transaminases > 2.5 ULN
Serum bilirubin > 1.5 ULN
Cirrhosis
Active viral hepatitis
Abdominal or hepatic irradiation
Previous use of gemtuzumab ozogamicin or inotuzumab ozogamicin
Hepatotoxic drugs
Iron overload

*HLA* human leukocyte antigen, *TBI* total body irradiation, *ULN* upper limit of normal

## How to treat

Some authors have reported on the use of high dose of methylprednisolone for SOS/VOD treatment. A prospective trial evaluated its administration at 0.5 mg/kg every 12 h during 7 days in 48 patients with SOS/VOD after allo-HCT, including 31% of patients with multiorgan failure (MOF) [23]. Thirty (63%) patients responded with a 50% or higher reduction in total serum bilirubin after 10 days of treatment. Fifty-eight percent of patients were alive at day +100 post allo-HCT. A second study retrospectively evaluated the use of methylprednisolone at 500 mg/m^2^ every 12 h for six doses in nine pediatric patients (including eight with MOF) [24]. Six responded to the treatment (≥50% reduction in bilirubin level after 10 days of treatment), but four of them also received treatment with DF. The same group subsequently reported the outcome of 15 additional HCT pediatric patients with SOS and treated with a combination high-dose prednisone and DF [25]. SOS/VOD complete resolution rate was 67%, with 73% of patients alive at day +100. Overall, data are scarce and difficult to interpret, mainly retrospective and single center, with no dose defined. We therefore recommend against the use of methylprednisolone alone as a prolonged primary treatment of SOS/VOD, especially given the risk of infectious complications associated with high-dose corticosteroids.

DF is the only agent with proven efficacy for the treatment of severe/very severe SOS/VOD. DF is a polydisperse oligonucleotide with antithrombotic, anti-ischemic, and anti-inflammatory activity at the level of the microvasculature [1, 26]. Although its precise mechanism of action in SOS/VOD remains an area of active investigation, it involves two distinct elements: the protection of endothelial cells and restoration of the thrombotic-fibrinolytic balance [1]. Over the past decade, multiple studies have evaluated the use of DF for SOS/VOD treatment (Table 4). A pivotal multicenter phase III trial assessed the effect of a 25 mg/kg/day dose in 102 patients (median age 21 years, range 0–72) with severe SOS/VOD [27]. For ethical reasons, a randomization with placebo or supportive care was not possible. Therefore, a historical control group (*n* = 32) was used in this trial. Therefore, a contemporaneous and rigorously defined historical control group was used in this trial (*n* = 32), using a novel methodology screening almost 7000 sequential patients. Treatment with DF was associated with a significantly higher CR rate (24 vs. 9%, *p* = 0.013) and day +100 OS (38 vs. 25%, *p* = 0.034). No differences in adverse event incidence were reported between the two groups, including for hemorrhagic toxicity (65 vs. 69%).

**Table 4:** Main studies on defibrotide in SOS/VOD

Reference; Phase; Number of patients	Condition	Design	CR rate	Others results
Richardson et al. [58] Retrospective CUP *N* = 19	Adult and pediatric Severe SOS post HCT	Compassionate use; DF: 5–60 mg/kg/day (individual patient dose escalation, until response/toxicity)	CR: 42% Minimal toxicity at doses tested	Day +100 survival: 32%
Richardson et al. [59] Phase I/II *N* = 88	Adult and pediatric Severe SOS post HCT	Emergency use; DF: 5–60 mg/kg/day (intrapatient dose escalation, until response/toxicity)	CR: 36% Active dose range 25–40 mg/kg/day	Day +100 survival: 35% No serious AEs attributed to DF
Richardson et al. [30] Phase II *N* = 149	Adult and pediatric Severe SOS post HCT	Randomized, dose-finding; Arm A: DF 25 mg/kg/day Arm B: DF 40 mg/kg/day For 14 days or more	Day +100 CR: 46% Effective dose 25 mg/kg/day	Day +100 survival: 42% Overall SAE incidence: 8% (greater at 40 vs. 25 mg/kg/day)
Richardson et al. [27] Phase III *N* = 102	Adult and pediatric Severe SOS post HCT	Nonrandomized, comparison to historical control; DF: 6.25 mg/kg IV q6h (25 mg/kg/day) for 21 days or more	Day +100 CR DF 24% HC 9% (*p* = 0.0131)	Day +100 mortality: DF 62%; HC 75% (*p* = 0.0341) Hemorrhagic AEs: DF 65%; HC 69%
Corbacioglu et al. [28] Compassionate use program *N* = 710	Adult and pediatric <18 years (*N* = 303) ≥18 years (*N* = 407) SOS non-HCT (*N* = 68) SOS post HCT (*N* = 628) Severe SOS (*N* = 292)	Investigational new drug protocol; DF: 10–80 mg/kg /day (median 25 mg/kg/day) for a median of 15 days (range, 1–119)	Day +100 CR Non-HCT 40% SOS/VOD post HCT 47% Severe SOS/VOD post HCT 29%	Day +100 survival: Pediatric: 69% Adults: 49% Severe SOS: 41% Nonsevere SOS: 68% Overall hemorrhagic AEs: 12%
Kernan et al. [29] Prospective T-IND *N* = 1000 (non-SCT excluded)	Adult and pediatric ≤16 years (*N* = 570) >16 years (*N* = 430) Severe SOS (*N* = 512)	Investigational new drug protocol; DF: 6.25 mg/kg IV q6h (25 mg/kg/day) for 21 days or more		Day +100 survival: 58.9% Pediatric: 67.9% Adults: 47.1% Severe SOS: 49.5% Nonsevere SOS: 68.9% Overall hemorrhagic AEs: 29%
Corbacioglu et al. [51] Phase III *N* = 356	Pediatric SOS/VOD prophylaxis post HCT	Randomized comparison; DF: 6.25 mg/kg IV q6h (25 mg/kg/day) from start conditioning to 30 days post HCT (at least 14 days if discharge before) Control: cross over to the DF arm in case of SOS/VOD onset	SOS/VOD incidence: DF 12% Control 20% *p* = 0.0488	Day +100 SOS/VOD related mortality: DF 2%, control 6%, *p* = 0.10 No difference in AEs and hemorrhagic AEs

*SOS* sinusoidal obstruction syndrome, *CUP* compassionate use program, *HSCT* hematopoietic stem cell transplantation, *DF* defibrotide, *CR* complete remission, *AE* adverse event

A large European compassionate use program included 407 adult patients (≥18 years old) with a day +100 OS of 49%, and an overall incidence of hemorrhagic events of 12% [28]. Similarly, prospective data from the large US expanded-access treatment protocol reported a day +100 OS of 47.1% among 430 adult patients (>16 years), and an overall incidence of hemorrhagic events of 29% [29].

The dose of 25 mg/kg/day is well established. A well-sized, multicenter phase II prospective study compared this (25 mg/kg/day, *n* = 75) with a higher dose (40 mg/kg/day, *n* = 74), without any difference in terms of CR rate (49 vs. 43%, *p* = 0.61) and OS at day +100 (44 vs. 39%; *p* = 0.62) [30]. Furthermore, a trend toward more toxicity was reported in the 40 mg/kg/day group, leading to the selection for the 25 mg/kg/day dose. In the compassionate use program, DF doses ranged from 10 to 80 mg/kg/day [28]. Day +100 OS was 43, 58, and 61% in patients receiving 10, 25, or 60/80 mg/kg/day, respectively. Importantly, DF at 25 mg/kg/day was associated with a higher OS compared with 10 mg/kg/day, while the difference was not significant compared with 60/80 mg/kg/day DF. Overall, the use of doses over 25 mg/kg/day seems to be associated with more toxicity without any clinical benefit, while lower doses are less effective. Therefore, the dose approved by the FDA and the EMA, and which we recommend, is 25 mg/kg/day. In patients with renal failure, no dose adjustments are required, while, in obese patients, corrected body weight should be used for dose calculation. The recommended duration of DF treatment is at least 21 days, and until resolution of all SOS/VOD symptoms. However, in patients where such resolution happens before 21 days of treatment, it is possible to stop DF earlier, in particular to facilitate patient’s discharge, but close monitoring is recommended as recurrence may rarely develop. Specifically, after completion of DF treatment and resolution of SOS/VOD symptoms, some cases of recurrence of SOS/VOD can be observed, albeit rarely. DF should be resumed at the same dose, and as clinically indicated, hepatic biopsy is recommended to rule out confounding alternate the diagnoses. So far, there are no data to support recommendations for any kind of maintenance treatment in these patients upon resolution of the second episode of SOS/VOD, although a course of at least 14–21 days of therapy would seem prudent.

Finally, physicians should also keep in mind the very small risk of anaphylaxis associated with DF [31].

## When to treat

Given the mortality associated with severe and very severe SOS/VOD, it is mandatory to treat these patients promptly, and DF should be initiated as soon as possible. The EBMT severity grading classification has facilitated the earlier identification of those patients [7]. The indication of DF treatment in patients with mild or moderate SOS/VOD is more questionable. However, several lines of evidence encourage this approach. In expanded-access treatment protocols, an earlier treatment initiation after SOS/VOD diagnosis was associated with higher day +100 OS (*p* < 0.001) [29, 32]. This suggests that DF, despite its relatively high cost, should be initiated immediately after diagnosis of SOS/VOD, rather than being delayed until the severity criteria assessment has been reached. Furthermore, in the compassionate use program and the expanded-access treatment protocol [29], the day +100 OS in the so-called nonsevere patients with DF therapy [28] was 68% and 68.9%, respectively, with an up to 32% mortality seen despite treatment. While these results compare favorably to patients with severe/very severe SOS/VOD, the mortality remains significant, highlighting the importance to treat these patients. Therefore, we recommend that patients who fulfill the EBMT diagnosis criteria for SOS/VOD [7] and whose severity grading is moderate should be considered for preemptive DF and closely followed. In patients with mild SOS/VOD, supportive care (see below) should be intensified, and severity criteria monitoring should be strictly applied to allow immediate DF initiation in case of deterioration.

## Supportive care

Cautious fluid and sodium balance management is recommended [1]. Importantly, renal function must be closely monitored with daily creatinine assessment, monitoring of ingesta-excreta, and twice daily weight measurement. Diuretic (furosemide and/or spironolactone) may be cautiously administered in some patients, as part of fluid balance control [1]. Massive ascites and/or pleural effusion may cause major discomfort or restrictive pulmonary syndrome, and symptomatic treatment may include oxygen therapy or drainage [1, 33]. The latter should be performed with the same precautions as any invasive procedure, and in particular for DF administration and platelet transfusion support. In patients with severe renal dysfunction, hemodialysis/hemofiltration is required [1]. Patients with severe MOD and MOF are generally transferred to an intensive care unit. The usefulness of transjugular intrahepatic portosystemic shunt is limited to symptomatic control, with no benefit on survival [34]. Cases of liver transplant in patients with severe SOS/VOD have been reported [35].

Elementary measures such as comfortable positioning, appropriate reassurance, and psychological support are also an important part in supportive care as well as during treatment. Pain can also result from massive ascites effusion, and its management is important for the patient’s comfort. If required, opioid can be carefully used taking into account the patient’s renal, hepatic, and pulmonary condition.

Nutritional support is also important, and enteral nutrition should be favored to prevent patient’s malnutrition. Parenteral nutrition is associated with fluid overload, infectious complications, and hepatotoxicity, and should be avoided. Furthermore, discontinuation of any other potential hepatotoxic drug should be discussed as to its risk/benefit ratio. In particular, whenever possible, antifungal azoles should be substituted for echinocandin. Ursodiol if not already administered, should be considered.

### Management of hemorrhagic risk in patients treated with DF

Given the hemorrhagic events reported in patients treated with DF, it is recommended to discontinue any other agents that may increase the risk of bleeding. This includes anticoagulants, but also any other drugs that have been associated with an increased risk of bleeding, such as ibrutinib [36]. The risk–benefit ratio must be carefully weighed, depending on the indication of the treatment. Furthermore, for every patient treated with DF, the threshold for platelet transfusion should be increased at 30 × 10^9^/L. We acknowledge that this threshold may not be achievable, particularly in the case of platelet refractoriness. For invasive procedures, in addition to platelet transfusions, DF should be suspended at least 2 h before and 2 h after the procedure, given its relatively short half-life (<2 h). For patients with life-threatening bleeding, DF must be immediately discontinued, and its resumption should be discussed on a case per case basis and according to the risk/benefit ratio. Although bleeding is usually not life threatening in patients with hemorrhagic cystitis or severe mucositis, their management may be difficult, and DF discontinuation may be necessary, again depending on the risk/benefit ratio. Fresh frozen plasma can be useful in some patients to correct the hemostasis disorder.

## Preventive therapy

### Non-pharmacologic measure

Non-pharmacologic measures rely on the reduction of SOS/VOD risk factors. However, patient-related and hepatic risk factors are often impossible to reverse. Nonetheless, it may be advisable to consider delaying the HCT, when feasible according to the disease status, until resolution or treatment of certain feature (such as iron overload and acute hepatitis) [1]. Furthermore, hepatotoxic drugs should be discontinued whenever possible [1].

In contrast, it may be easier to modify transplant-related risk factors through optimization of the conditioning regimen. For example, RIC regimen should be preferred in older patients or those with comorbidities. Reduced toxicity conditioning regimens based on fludarabine and IV busulfan, and so avoiding high-dose cyclophosphamide or total body irradiation, are also effective. In addition, in vivo T-cell depletion, especially in the case of HLA-mismatched donors, is recommended. Finally, for graft-vs.-host prophylaxis, the combination of sirolimus with a calcineurin inhibitor should be avoided if SOS/VOD is a concern, and cyclosporine-A should be substituted for another calcineurin inhibitor [7, 37].

### Pharmacologic measures

Heparins have previously been used for SOS/VOD prophylaxis. However, a large meta-analysis reported that the use of unfractionated heparin or low-molecular-weight heparin prophylaxis was not associated with a significant decrease in the risk of SOS/VOD (pooled relative risk, 0.90; 95% confidence interval, 0.62–1.29) [38]. Furthermore, bleeding was reported as an adverse event in 7 of the 12 studies under the meta-analysis (2782 patients) [38]. Therefore, given the absence of conclusive results on its effectiveness and its potential side effects, heparin should be abandoned as SOS prophylaxis.

Other agents have been evaluated for SOS/VOD prophylaxis, including anti-thrombin, prostaglandin E1, or pentoxifylline. However, none of these agents showed efficacy for SOS/VOD prevention, and some were associated with severe side effects [39–44]. Therefore, they are not recommended for prevention of SOS/VOD.

Ursodeoxycholic acid for SOS/VOD prevention has been evaluated in several prospective randomized trials. Some demonstrated a decreased incidence of SOS/VOD [45, 46], while others failed to uncover an advantage [47, 48]. However, the combined results of the three prospective clinical trials using ursodeoxycholic acid alone as prophylaxis vs. no treatment demonstrated a reduced proportion of SOS (relative risk, 0.34; 95% confidence interval, 0.17–0.66) [49]. Furthermore, continuous administration of ursodeoxycholic acid until 90 days after transplantation significantly reduced the proportion of patients developing high serum bilirubin levels, severe acute GVHD, liver GVHD, and intestinal GVHD, translating into a significantly lower non-relapse mortality and better OS [50]. Therefore, the use of ursodeoxycholic acid is recommended from the beginning of the conditioning until day 90 after transplantation.

Prophylaxis of SOS/VOD with DF has been evaluated in a randomized prospective phase III study in 356 pediatric patients at high risk of developing SOS/VOD [51]. All patients received a myeloablative conditioning regimen HCT and had one or more risk factors for SOS/VOD. In the prophylaxis arm (*n* = 180), DF was administered at 25 mg/kg per day from day 1 of the conditioning until day 30 post HCT, while in the control arm (*n* = 176) no prophylactic treatment was administered. In the control arm, a crossover approach allowed patients to receive DF when they developed SOS/VOD (according to the modified Seattle criteria). Prophylactic DF was associated with a significantly reduced incidence of SOS/VOD; specifically, 12 vs. 20% in the control arm (*p* = 0.048), the trial’s primary endpoint. This did not translate into a reduction of day 100 SOS/VOD-associated mortality (2 vs. 6%, *p* = 0.10), not least due to the crossover design, and the limited power of the trial to establish this secondary endpoint. However, development of SOS/VOD was associated with a four-time higher mortality (25 vs. 6%, *p* < 0.0001) overall, and there was no difference in the incidence of adverse events, including hemorrhage, between the two groups.

In adult patients, no prospective study has yet reported on DF for SOS/VOD prevention. However, several retrospective studies have described a very low incidence of SOS/VOD with DF prophylaxis (ranging from 0 to 2%) in heterogeneous cohort of adult patients undergoing either autologous or allo-HCT [52–54]. Recently, DF prophylaxis was assessed in a cohort of 63 adult patients treated with allo-HCT and considered at high risk for SOS [55]. DF was generally well tolerated, with only four patients (6%) discontinuing DF because of bleeding events, and the median duration of treatment was 23 days. Four patients (6%) developed SOS/VOD, two within 21 days, and two beyond day 21 (late-onset SOS/VOD). A prospective randomized confirmatory trial in adults and children is underway (NCT02851407).

Overall, despite the absence of published randomized clinical trial evaluating DF for SOS/VOD prophylaxis, based on the benefit observed in the randomized pediatric clinical trial and on retrospective results available in adults, DF prophylaxis can be considered in adult patients at very high risk of SOS/VOD. Very high-risk patients are defined by the presence of at least two major risk factors: previous treatment with gemtuzumab ozogamicin or inotuzumab, established liver disease, conditioning regimen with three or more alkylating agents (including posttransplant cyclophosphamide), or with a high dose of total body irradiation and an alkylating agent, previous liver irradiation, and previous allogeneic or autologous HCT (excluding autologous HCT with high-dose melphalan for multiple myeloma) [1, 56]. In patients with only one major and/or multiple minor risk factors, there is no literature supporting the use of DF for SOS/VOD prophylaxis. Alternatively, the CIBMTR recently published a risk score identifying patients at high risk for VOD could be used [57]. This risk score take into account patient’s age, Karnofsky, sirolimus use, hepatitis B/C status, conditioning regimen, and disease/disease status at time of transplantation (link for calculation https://www.cibmtr.org/ReferenceCenter/Statistical/Tools/Pages/VOD.aspx).

DF prophylaxis should be initiated with the start of conditioning, and administered until day 21 or earlier, in the case of earlier patient discharge. The drug should be administered at the same dose as in the therapeutic setting (25 mg/kg per day divided in four daily doses of 6.25 mg/kg). In the case of SOS/VOD development under prophylaxis, DF should be administered at the same dose, as there is no reason to increase it, and maintained for 21 days after the diagnosis of SOS/VOD.

## Future considerations

By providing these recommendations for SOS/VOD prevention and treatment, the aim is to standardize the approach and improve patients’ outcome after HCT. However, besides HCT, new concerns are emerging with the development of new treatments. For example, the high incidence of SOS/VOD associated with inotuzumab raises the question of SOS/VOD careful monitoring in this setting, in particular as to the role of DF prophylaxis. Similarly, the landscape of immunotherapy is rapidly evolving with the development of CAR T cells and other strategies associated with vascular injury. We must therefore be particularly cautious regarding the potential for additive toxicity of these treatments and, possible, SOS/VOD, especially when combined with HCT.

Another important parameter to be considered is added cost, the risk–benefit ratio and cost effectiveness of prophylactic or preemptive DF in the context of HCT. Such evaluation should be performed, preferably in a prospective trial or through capturing real-world data generated, e.g., within the EBMT registry in close collaboration with health technology assessment bodies, to evaluate the impact and the relevance of our recommendations, and with overarching goal of improving patient outcome.

## References

[1] Mohty M, Malard F, Abecassis M, Aerts E, Alaskar A S, Aljurf M, Arat M, Bader P, Baron F, Bazarbachi A, Blaise D, Ciceri F, Corbacioglu S, Dalle J-H, Duarte R F, Fukuda T, Huynh A, Masszi T, Michallet M, Nagler A, NiChonghaile M, Pagluica T, Peters C, Petersen F B, Richardson P G, Ruutu T, Savani B N, Wallhult E, Yakoub-Agha I, Carreras E (2015). Sinusoidal obstruction syndrome/veno-occlusive disease: current situation and perspectives—a position statement from the European Society for Blood and Marrow Transplantation (EBMT). Bone Marrow Transplantation.

[2] Carreras E, Diaz-Ricart M (2011). The role of the endothelium in the short-term complications of hematopoietic SCT. Bone Marrow Transpl.

[3] McDonald GB, Sharma P, Matthews DE, Shulman HM, Thomas ED (1984). Venocclusive disease of the liver after bone marrow transplantation: diagnosis, incidence, and predisposing factors. Hepatology.

[4] Jones RJ, Lee KS, Beschorner WE, Vogel VG, Grochow LB, Braine HG (1987). Venoocclusive disease of the liver following bone marrow transplantation. Transplantation.

[5] Coppell JA, Richardson PG, Soiffer R, Martin PL, Kernan NA, Chen A (2010). Hepatic veno-occlusive disease following stem cell transplantation: incidence, clinical course, and outcome. Biol Blood Marrow Transplant.

[6] McDonald GB, Hinds MS, Fisher LD, Schoch HG, Wolford JL, Banaji M (1993). Veno-occlusive disease of the liver and multiorgan failure after bone marrow transplantation: a cohort study of 355 patients. Ann Intern Med.

[7] Mohty M, Malard F, Abecassis M, Aerts E, Alaskar A S, Aljurf M, Arat M, Bader P, Baron F, Bazarbachi A, Blaise D, Ciceri F, Corbacioglu S, Dalle J-H, Dignan F, Fukuda T, Huynh A, Masszi T, Michallet M, Nagler A, NiChonghaile M, Okamoto S, Pagliuca A, Peters C, Petersen F B, Richardson P G, Ruutu T, Savani B N, Wallhult E, Yakoub-Agha I, Duarte R F, Carreras E (2016). Revised diagnosis and severity criteria for sinusoidal obstruction syndrome/veno-occlusive disease in adult patients: a new classification from the European Society for Blood and Marrow Transplantation. Bone Marrow Transplantation.

[8] Carreras Enric (2014). How I manage sinusoidal obstruction syndrome after haematopoietic cell transplantation. British Journal of Haematology.

[9] Carreras E, Granena A, Navasa M, Bruguera M, Marco V, Sierra J (1993). On the reliability of clinical criteria for the diagnosis of hepatic veno-occlusive disease. Ann Hematol.

[10] Yakushijin K, Atsuta Y, Doki N, Yokota A, Kanamori H, Miyamoto T (2016). Sinusoidal obstruction syndrome after allogeneic hematopoietic stem cell transplantation: Incidence, risk factors and outcomes. Bone Marrow Transpl.

[11] Carreras E, Rosinol L, Terol MJ, Alegre A, de Arriba F, Garcia-Larana J (2007). Veno-occlusive disease of the liver after high-dose cytoreductive therapy with busulfan and melphalan for autologous blood stem cell transplantation in multiple myeloma patients. Biol Blood Marrow Transpl.

[12] Lee JL, Gooley T, Bensinger W, Schiffman K, McDonald GB (1999). Veno-occlusive disease of the liver after busulfan, melphalan, and thiotepa conditioning therapy: incidence, risk factors, and outcome. Biol Blood Marrow Transpl.

[13] Yoon Jae-Ho, Yoo Keon Hee, Sung Ki Woong, Jung Chul Won, Kim Jin Seok, Hahn Seung Min, Kang Hyoung Jin, Lee Je-Hwan, Im Ho Joon, Ahn Jae-Sook, Kook Hoon, Cho Bin, Lee Jong Wook (2019). Validation of treatment outcomes according to revised severity criteria from European Society for Blood and Marrow Transplantation (EBMT) for sinusoidal obstruction syndrome/veno-occlusive disease (SOS/VOD). Bone Marrow Transplantation.

[14] Bashey A, Zhang MJ, McCurdy SR, St Martin A, Argall T, Anasetti C (2017). Mobilized peripheral blood stem cells versus unstimulated bone marrow as a graft source for T-cell-replete haploidentical donor transplantation using post-transplant cyclophosphamide. J Clin Oncol.

[15] Yu X, Liu L, Xie Z, Dong C, Zhao L, Zhang J (2019). Bone marrow versus peripheral blood as a graft source for haploidentical donor transplantation in adults using post-transplant cyclophosphamide-A systematic review and meta-analysis. Crit Rev Oncol/Hematol.

[16] Kantarjian HM, DeAngelo DJ, Stelljes M, Martinelli G, Liedtke M, Stock W (2016). Inotuzumab ozogamicin versus standard therapy for acute lymphoblastic leukemia. N Engl J Med.

[17] Toksvang LN, De Pietri S, Nielsen SN, Nersting J, Albertsen BK, Wehner PS, et al. Hepatic sinusoidal obstruction syndrome during maintenance therapy of childhood acute lymphoblastic leukemia is associated with continuous asparaginase therapy and mercaptopurine metabolites. Pediatr Blood Cancer. 2017; 64. 10.1002/pbc.26519 28423235

[18] Johnson DB, Chandra S, Sosman JA (2018). Immune checkpoint inhibitor toxicity in 2018. JAMA.

[19] Ijaz A, Khan AY, Malik SU, Faridi W, Fraz MA, Usman M (2019). Significant Risk of graft-versus-host disease with exposure to checkpoint inhibitors before and after allogeneic transplantation. Biol Blood Marrow Transpl.

[20] Haverkos BM, Abbott D, Hamadani M, Armand P, Flowers ME, Merryman R, et al. PD-1 blockade for relapsed lymphoma post allogeneic hematopoietic cell transplant: high response rate but frequent GVHD. Blood. 2017;130:221–8. 10.1182/blood-2017-01-761346 PMC5510790 28468799

[21] Merryman RW, Kim HT, Zinzani PL, Carlo-Stella C, Ansell SM, Perales MA (2017). Safety and efficacy of allogeneic hematopoietic stem cell transplant after PD-1 blockade in relapsed/refractory lymphoma. Blood.

[22] Herbaux C, Gauthier J, Brice P, Drumez E, Ysebaert L, Doyen H (2017). Efficacy and tolerability of nivolumab after allogeneic transplantation for relapsed Hodgkin lymphoma. Blood.

[23] Al Beihany A, Al Omar H, Sahovic E, Chaudhri N, Al Mohareb F, Al Sharif F (2008). Successful treatment of hepatic veno-occlusive disease after myeloablative allogeneic hematopoietic stem cell transplantation by early administration of a short course of methylprednisolone. Bone Marrow Transpl.

[24] Myers KC, Lawrence J, Marsh RA, Davies SM, Jodele S (2013). High-dose methylprednisolone for veno-occlusive disease of the liver in pediatric hematopoietic stem cell transplantation recipients. Biol Blood Marrow Transpl.

[25] Gloude NJ, Jodele S, Teusink-Cross A, Grimley M, Davies SM, Lane A (2018). Combination of high-dose methylprednisolone and defibrotide for veno-occlusive disease in pediatric hematopoietic stem cell transplant recipients. Biol Blood Marrow Transpl.

[26] Richardson PG, Corbacioglu S, Ho VT, Kernan NA, Lehmann L, Maguire C (2013). Drug safety evaluation of defibrotide. Expert Opin Drug Saf.

[27] Richardson Paul G., Riches Marcie L., Kernan Nancy A., Brochstein Joel A., Mineishi Shin, Termuhlen Amanda M., Arai Sally, Grupp Stephan A., Guinan Eva C., Martin Paul L., Steinbach Gideon, Krishnan Amrita, Nemecek Eneida R., Giralt Sergio, Rodriguez Tulio, Duerst Reggie, Doyle John, Antin Joseph H., Smith Angela, Lehmann Leslie, Champlin Richard, Gillio Alfred, Bajwa Rajinder, D’Agostino Ralph B., Massaro Joseph, Warren Diane, Miloslavsky Maja, Hume Robin L., Iacobelli Massimo, Nejadnik Bijan, Hannah Alison L., Soiffer Robert J. (2016). Phase 3 trial of defibrotide for the treatment of severe veno-occlusive disease and multi-organ failure. Blood.

[28] Corbacioglu S, Carreras E, Mohty M, Pagliuca A, Boelens JJ, Damaj G (2016). Defibrotide for the treatment of hepatic veno-occlusive disease: final results from the international compassionate-use program. Biol Blood Marrow Transpl.

[29] Kernan NA, Grupp S, Smith AR, Arai S, Triplett B, Antin JH (2018). Final results from a defibrotide treatment-IND study for patients with hepatic veno-occlusive disease/sinusoidal obstruction syndrome. Br J Haematol.

[30] Richardson PG, Soiffer RJ, Antin JH, Uno H, Jin Z, Kurtzberg J (2010). Defibrotide for the treatment of severe hepatic veno-occlusive disease and multiorgan failure after stem cell transplantation: a multicenter, randomized, dose-finding trial. Biol Blood Marrow Transpl.

[31] Artesani MC (2006). Anaphylactic shock to defibrotide. Allergy.

[32] Richardson PG, Smith AR, Triplett BM, Kernan NA, Grupp SA, Antin JH (2017). Earlier defibrotide initiation post-diagnosis of veno-occlusive disease/sinusoidal obstruction syndrome improves day +100 survival following haematopoietic stem cell transplantation. Br J Haematol.

[33] Mashegu H, Smith L, Li Y, Seif A, Grupp S, Bunin N (2018). The role of peritoneal drainage in veno-occlusive disease in pediatric patients post hematopoietic stem cell transplant. Bone Marrow Transpl.

[34] Azoulay D, Castaing D, Lemoine A, Hargreaves GM, Bismuth H (2000). Transjugular intrahepatic portosystemic shunt (TIPS) for severe veno-occlusive disease of the liver following bone marrow transplantation. Bone Marrow Transpl.

[35] Kim ID, Egawa H, Marui Y, Kaihara S, Haga H, Lin YW (2002). A successful liver transplantation for refractory hepatic veno-occlusive disease originating from cord blood transplantation. Am J Transpl.

[36] Stephens Deborah M., Byrd John C. (2019). How I manage ibrutinib intolerance and complications in patients with chronic lymphocytic leukemia. Blood.

[37] Carmona A, Diaz-Ricart M, Palomo M, Molina P, Pino M, Rovira M (2013). Distinct deleterious effects of cyclosporine and tacrolimus and combined tacrolimus-sirolimus on endothelial cells: protective effect of defibrotide. Biol Blood Marrow Transpl.

[38] Imran H, Tleyjeh IM, Zirakzadeh A, Rodriguez V, Khan SP (2006). Use of prophylactic anticoagulation and the risk of hepatic veno-occlusive disease in patients undergoing hematopoietic stem cell transplantation: a systematic review and meta-analysis. Bone Marrow Transpl.

[39] Haussmann U, Fischer J, Eber S, Scherer F, Seger R, Gungor T (2006). Hepatic veno-occlusive disease in pediatric stem cell transplantation: impact of pre-emptive antithrombin III replacement and combined antithrombin III/defibrotide therapy. Haematologica.

[40] Bearman SI, Shen DD, Hinds MS, Hill HA, McDonald GB (1993). A phase I/II study of prostaglandin E1 for the prevention of hepatic venocclusive disease after bone marrow transplantation. Br J Haematol.

[41] Bordigoni P, Witz F, Von Bueltzingsloewen A, Schmitt C, Sommelet D (1991). Prostaglandin E1 (PGE1) induced arthritis following bone marrow transplantation. Br J Haematol.

[42] Song JS, Seo JJ, Moon HN, Ghim T, Im HJ (2006). Prophylactic low-dose heparin or prostaglandin E1 may prevent severe veno-occlusive disease of the liver after allogeneic hematopoietic stem cell transplantation in Korean children. J Korean Med Sci.

[43] Attal M, Huguet F, Rubie H, Charlet JP, Schlaifer D, Huynh A (1993). Prevention of regimen-related toxicities after bone marrow transplantation by pentoxifylline: a prospective, randomized trial. Blood.

[44] Clift RA, Bianco JA, Appelbaum FR, Buckner CD, Singer JW, Bakke L (1993). A randomized controlled trial of pentoxifylline for the prevention of regimen-related toxicities in patients undergoing allogeneic marrow transplantation. Blood.

[45] Essell JH, Schroeder MT, Harman GS, Halvorson R, Lew V, Callander N (1998). Ursodiol prophylaxis against hepatic complications of allogeneic bone marrow transplantation. A randomized, double-blind, placebo-controlled trial. Ann Intern Med.

[46] Ohashi K, Tanabe J, Watanabe R, Tanaka T, Sakamaki H, Maruta A (2000). The Japanese multicenter open randomized trial of ursodeoxycholic acid prophylaxis for hepatic veno-occlusive disease after stem cell transplantation. Am J Hematol.

[47] Park SH, Lee MH, Lee H, Kim HS, Kim K, Kim WS (2002). A randomized trial of heparin plus ursodiol vs. heparin alone to prevent hepatic veno-occlusive disease after hematopoietic stem cell transplantation. Bone Marrow Transpl.

[48] Ruutu T, Eriksson B, Remes K, Juvonen E, Volin L, Remberger M (2002). Ursodeoxycholic acid for the prevention of hepatic complications in allogeneic stem cell transplantation. Blood.

[49] Tay J, Tinmouth A, Fergusson D, Huebsch L, Allan DS (2007). Systematic review of controlled clinical trials on the use of ursodeoxycholic acid for the prevention of hepatic veno-occlusive disease in hematopoietic stem cell transplantation. Biol Blood Marrow Transpl.

[50] Ruutu T, Juvonen E, Remberger M, Remes K, Volin L, Mattsson J (2014). Improved survival with ursodeoxycholic acid prophylaxis in allogeneic stem cell transplantation: long-term follow-up of a randomized study. Biol Blood Marrow Transpl.

[51] Corbacioglu S, Cesaro S, Faraci M, Valteau-Couanet D, Gruhn B, Rovelli A (2012). Defibrotide for prophylaxis of hepatic veno-occlusive disease in paediatric haemopoietic stem-cell transplantation: an open-label, phase 3, randomised controlled trial. Lancet.

[52] Chalandon Y, Roosnek E, Mermillod B, Newton A, Ozsahin H, Wacker P (2004). Prevention of veno-occlusive disease with defibrotide after allogeneic stem cell transplantation. Biol Blood Marrow Transpl.

[53] Dignan F, Gujral D, Ethell M, Evans S, Treleaven J, Morgan G (2007). Prophylactic defibrotide in allogeneic stem cell transplantation: minimal morbidity and zero mortality from veno-occlusive disease. Bone Marrow Transpl.

[54] Park M, Park HJ, Eom HS, Kwon YJ, Park JA, Lim YJ (2013). Safety and effects of prophylactic defibrotide for sinusoidal obstruction syndrome in hematopoietic stem cell transplantation. Ann Transplant.

[55] Picod A, Bonnin A, Battipaglia G, Giannotti F, Ruggeri A, Brissot E (2018). Defibrotide for sinusoidal obstruction syndrome/veno-occlusive disease prophylaxis in high-risk adult patients: a single-center experience study. Biol Blood Marrow Transpl.

[56] Dalle JH, Giralt SA (2016). Hepatic veno-occlusive disease after hematopoietic stem cell transplantation: risk factors and stratification, prophylaxis, and treatment. Biol Blood Marrow Transpl.

[57] Strouse C, Zhang Y, Zhang MJ, DiGilio A, Pasquini M, Horowitz MM (2018). Risk Score for the development of veno-occlusive disease after allogeneic hematopoietic cell transplant. Biol Blood Marrow Transpl.

[58] Richardson PG, Elias AD, Krishnan A, Wheeler C, Nath R, Hoppensteadt D (1998). Treatment of severe veno-occlusive disease with defibrotide: compassionate use results in response without significant toxicity in a high-risk population. Blood.

[59] Richardson PG, Murakami C, Jin Z, Warren D, Momtaz P, Hoppensteadt D (2002). Multi-institutional use of defibrotide in 88 patients after stem cell transplantation with severe veno-occlusive disease and multisystem organ failure: response without significant toxicity in a high-risk population and factors predictive of outcome. Blood.

